# Identification and Functional Analysis of Three Isoforms of Bovine BST-2

**DOI:** 10.1371/journal.pone.0041483

**Published:** 2012-07-20

**Authors:** Eri Takeda, So Nakagawa, Yuki Nakaya, Atsushi Tanaka, Takayuki Miyazawa, Jiro Yasuda

**Affiliations:** 1 Department of Emerging Infectious Diseases, Institute of Tropical Medicine, Nagasaki University, Nagasaki, Japan; 2 Center for Information Biology and DNA Data Bank of Japan (DDBJ), National Institute of Genetics, Mishima, Japan; 3 The Japan Society for the Promotion of Science, Tokyo, Japan; 4 Laboratory of Signal Transduction, Institute for Virus Research, Kyoto University, Kyoto, Japan; 5 Department of Virology and Preventive Medicine, Gunma University Graduate School of Medicine, Maebashi, Japan; Institut Pasteur, France

## Abstract

Human BST-2 (hBST-2) has been identified as a cellular antiviral factor that blocks the release of various enveloped viruses. Orthologues of BST-2 have been identified in several species, including human, monkeys, pig, mouse, cat and sheep. All have been reported to possess antiviral activity. Duplication of the BST-2 gene has been observed in sheep and the paralogues are referred to as ovine BST-2A and BST2-B, although only a single gene corresponding to BST-2 has been identified in most species. In this study, we identified three isoforms of bovine BST-2, named bBST-2A1, bBST-2A2 and bBST-2B, in bovine cells treated with type I interferon, but not in untreated cells. Both bBST-2A1 and bBST-2A2 are posttranslationally modified by *N*-linked glycosylation and a GPI-anchor as well as hBST-2, while bBST-2B has neither of these modifications. Exogenous expression of bBST-2A1 or bBST-2A2 markedly reduced the production of bovine leukemia virus and vesicular stomatitis virus from cells, while the antiviral activity of bBST-2B was much weaker than those of bBST-2A1 and bBST-2A2. Our data suggest that bBST-2A1 and bBST-2A2 function as part of IFN-induced innate immunity against virus infection. On the other hand, bBST-2B may have a different physiological function from bBST-2A1 and bBST-2A2.

## Introduction

Human BST-2 (hBST-2) (also referred to as Tetherin, CD317 or HM1.24) was first identified as a cellular restriction factor that blocks the release of HIV-1 in the absence of the viral accessory protein, Vpu [1], [2]. Subsequent studies have shown that hBST-2 also inhibits the production of many other enveloped viruses, including retroviruses, filoviruses, arenaviruses, herpesviruses and rhabdoviruses [1], [3], [4], [5], [6], [7], [8], [9]. Orthologues of BST-2 have been identified in several species, including monkeys, pig, mouse, cat and sheep [1], [2], [4], [10], [11], [12], [13], [14], [15], [16], [17], [18]. All have been reported to possess antiviral activity.

BST-2 consists of four domains, *i.e.*, an N-terminal cytoplasmic tail (CT), a single transmembrane domain, an extracellular domain and a C-terminal glycosylphosphatidyl inositol (GPI) anchor, and therefore both ends of this molecule are associated with the plasma membrane [19]. Both the N-terminal transmembrane domain and C-terminal GPI anchor are essential for the antiviral activity of hBST-2 against HIV-1 [20]. BST-2 appears to inhibit HIV-1 release by directly tethering virions to cells, briefly by anchoring one end of the molecule on the cell membrane and the other end on the viral envelope.

The extracellular domain of hBST-2 has two *N*-linked glycosylation sites, which are highly conserved at the same positions among human, rhesus monkey, dog, pig, mouse and rat [19], [21]. Previously, we showed that *N*-linked glycosylation is dispensable for the antiviral activity of hBST-2 against Lassa and Marburg viruses [7]. On the other hand, there are conflicting data regarding the role of *N*-linked glycosylation on the antiviral activity of hBST-2 against HIV-1. Andrew *et al.* reported that *N*-linked glycosylation is not important for inhibition of HIV-1 virus release, while Perez-Caballero *et al.* showed that *N*-linked glycosylation, especially at the second site, is important for the antiviral activity of hBST-2 against HIV-1 [20], [22].

BST-2 is broadly induced by treatment with type I interferons (IFNs) in various cell types [23], [24]. Therefore, BST-2 is thought to function as a host innate antiviral system against a wide variety of viruses. Recent *in vivo* analyses showed that hBST-2 was expressed to varying degrees in most organs and a number of specialised cell types, including hepatocytes, pneumocytes, ducts of major salivary glands, pancreas and kidney, Paneth cells, epithelia, Leydig cells, plasma cells, bone marrow stromal cells, monocytes and vascular endothelium, without IFN stimulation [25]. These observations suggest that IFN may only partially regulate BST-2 *in vivo* and that BST-2 may have other important functions *in viv*o in addition to its role in antiviral defence.

Recently, genetic analyses of genome data suggested duplication of the BST-2 gene in ruminants, including cattle and sheep. In fact, Arnaud *et al.* reported that there are two isoforms of BST-2 in sheep referred to as ovine BST-2A and BST2-B [18]. For cattle, it has been reported the molecular cloning of only one isoform of BST-2 and its antiviral activity against prototypic foamy virus [26]. Cattle are among the most important domestic animals and are often infected with viruses, such as foot-and-mouth disease virus and bovine viral diarrhoea virus, resulting in severe economic damage. Therefore, in this study, to investigate the involvement of BST-2 in antiviral host defence of cattle and the possibility that BST-2 has functions in addition to its antiviral effects, we cloned and characterised bovine BST-2 genes.

## Materials and Methods

### Cells

Madin–Darby bovine kidney (MDBK) cells (ATCC CCL-22) and Madin–Darby canine kidney (MDCK) cells (ATCC CCL-34) were maintained at 37°C in a 5% CO_2_ incubator in Minimal Essential Medium (GIBCO, Auckland, NZ) supplemented with 5% fetal bovine serum and penicillin/streptomycin (GIBCO). Human embryonic kidney (HEK) 293T cells (ATCC CRL-11268), HeLa cells (ATCC CCL-2), and fetal lamb kidney cells constitutively producing bovine leukemia virus (BLV), FLK-BLV cells [27], were maintained in Dulbecco's modified Eagle's medium (Sigma, St. Louis, MO, USA) supplemented with 10% fetal bovine serum and penicillin/streptomycin.

### Cloning and plasmid construction of bovine BST-2 genes

MDBK cells were treated for 24 h in the presence of 1,000 units (2.4 µg/mL) of IFN-αA/D (Sigma) and then total cellular RNA was extracted from cells using an RNeasy Mini kit (Qiagen, Valencia, CA, USA). Based on the genome data of *Bos taurus*, we designed the primer sets specific for bBST-2A gene (XM_871059 and XM_002688577), 5′-GGGGTACCGATGCACTACAGACCAGTGCCC-3′ and 5′-CCAAGCTTCAGGTCAGCAGAGCGTTGAGG-3′, or bBST-2B gene (XM_584000), 5′-GGGGTACCGATGGACACCGAGGAGGACATG-3′ and 5′-CCAAGCTTCATTTCTTCAGACACTTGCAGA-3′. RT-PCR was performed using a PrimeSTAR RT-PCR Kit (Takara, Shiga, Japan), with total RNA extracted from IFN-treated MDBK cells as the template, according to the manufacturer's protocols. In addition, bovine glyceraldehyde-3-phosphate dehydrogenase (GAPDH) gene was detected as an internal control gene in RT-PCR analysis by using the primer sets, 5′-GCCAAGGCTATCCATGACAACTTTGG-3′ and 5′-GGCGGCAGGCGATTCAGGCCTTGGAA-3′. The amplified bBST2 cDNAs were cloned into the *Kpn*I/*Hin*dIII digest of pCDNFL, which was previously constructed from pcDNA3.1 (Invitrogen, Carlsbad, CA, USA) to express a protein containing a FLAG-tag at the N-terminus [7], [28]. The expression plasmid for human BST-2, pTeth-FL, was described previously [7], [29].

### Genetic analysis

Three isoforms of bBST2 were mapped into the bovine genome (UMD 3.1 assembly) using the BLAT program [30]. Multiple amino acid sequence alignment was performed using L-INS-i in MAFFT [31] and modified it manually. Based on the amino acid alignment, nucleotides from each isoform were aligned using TranslatorX [32]. The amino acid substitution model of JTT with gamma-distributed rate variation (+Γ, α = 0.591) was selected using the Akaike information criterion implemented in PROTTEST 3 [33]. A phylogenetic tree was constructed using the maximum-likelihood method in RAxML v7.2.6 [34] with robustness evaluated by rapid bootstrapping (1,000 times).

### Western blotting analysis

293T cells were transfected with 1 µg of the expression plasmid for human or bovine BST-2 by using the TransIT-2020 Transfection Reagent (Mirus Bio Corp., Madison, WI, USA). At 24 h after transfection, cells were collected and once lysed with the SDS sample buffer (25 mM Tris HCl [pH 6.8], 5% Glycerol, 1% sodium dodecyl sulphate (SDS) and 5% 2-mercaptethanol). Cell lysates were electrophoresed on 12.5% SDS-polyacrylamide gels and then analyzed by Immunoblotting using anti-FLAG M2 and anti-Actin antibodies (Sigma-Aldrich, St. Louis, MO, USA). For *N*-glycosylation analysis, the buffer was changed to PBS (pH 7.4) by gel filtration and cell lysates were incubated with PNGase F (New England Biolabs, Ipswich, MA, USA) for 1 h at 37°C. After incubation, sample buffer was added and the lysates were subjected to Immunoblotting analysis as described above.

### Immunofluorescence microscopy

At 24 h posttransfection, MDBK cells and HeLa cells expressing FLAG-tagged BST-2 were fixed in 4% paraformaldehyde/PBS for longer than 1 h at 4°C and then treated with 0.2% Triton X-100 for 7 min. FLAG-tagged BST-2 or CD63 was stained with FITC-conjugated anti-FLAG M2 antibody (Sigma) or TRITC-conjugated anti-LAMP-3 antibody (CD63; Santa Cruz Biotechnology, Santa Cruz, CA, USA), respectively. Trans-Golgi compartments were stained with rabbit anti-TGN46 antibody (Abcam, Cambridge, UK), followed by goat anti-rabbit IgG conjugated with TRITC (Sigma). After staining of nuclei with DAPI (4′,6-diamino-2-phenylindole), cells were observed by fluorescence microscopy (BZ-8000; Keyence, Osaka, Japan) and confocal microscopy (LSM780 ELYRA system; Carl Zeiss, Oberkochen, Germany).

### Assay for antiviral activity of bBST-2s against bovine leukemia virus

FLK-BLV cells were electroporated with the expression plasmid for bBST-2A1, bBST-2A2, bBST-2B or hBST-2, or the empty vector, pCDNFL, using the Nucleofector electroporation system (Lonza, Basel, Switzerland). At 12 h post-electroporation, culture media were changed to fresh one and then cells were incubated again. After incubation for 24 h and 48 h, culture media were collected. To quantify the release of BLV from cells, BLV genomic RNAs were extracted from culture media using a QIAamp Viral RNA Mini Kit (Qiagen). After DNaseI treatment, real-time RT-PCR was performed using a One Step SYBR RT-PCR Kit (Takara) according to the manufacture's protocols. The primers targeting the BLV *tax* region (Accession No. EF600696), 5′-ACTGGACCGCCGATGGACGA-3′ (forward) and 5′-AAGACAGGCCGGGCGTTTGG-3′ (reverse), were used for real-time RT-PCR. The thermal profile was at 42°C for 5 min and 95°C for 10 s, followed by 40 cycles of 94°C for 5 s and 61°C for 20 s. Thermal cycling and quantification were performed using a Smart Cycler II System (Cepheid, Sunnyvale, CA). To generate the standard curve for cycle thresholds versus copy numbers, the vector containing targeting fragment was constructed by the insertion of the PCR fragment amplified by using the above primers into pCR4-TOPO vector using a TOPO-TA cloning Kit (Invitrogen) according to the manufacture's protocols.

### Assay for antiviral activity of bBST-2s against vesicular stomatitis virus

MDCK cells were electroporated with the expression plasmid for bBST-2A1, bBST-2A2, bBST-2B or hBST-2, or pCDNFL using the Nucleofector electroporation system, according to the manufacturer's protocols. At 24 h post-electroporation, cells were infected with vesicular stomatitis virus (VSV) Indiana strain at multiplicity of infection (MOI) of 0.001 for 1 h and then washed three times with PBS. Fresh MEM containing 0.5% fetal bovine serum was added to each well and cells were incubated for 12 h at 37°C. To quantify the progeny viruses produced from cells, culture media were collected and then titrated by plaque assay as reported previously [35], [36].

## Results

### Cloning and sequence analysis of bovine BST-2

**Figure 1:**
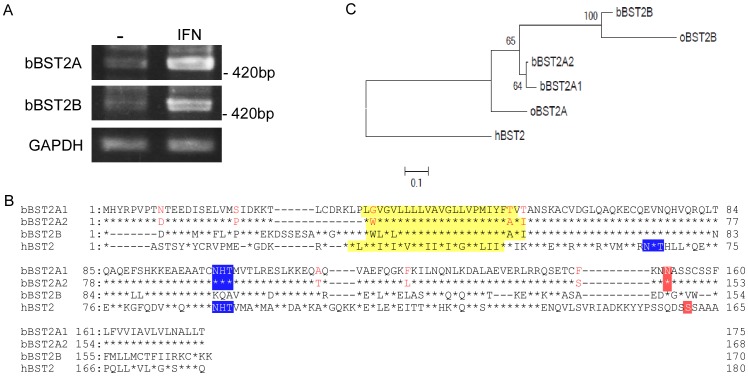
Deduced amino acid sequences of bovine BST-2s. (A) RT-PCR was carried out for RNA extracted from MDBK cells treated with or without IFN-α, using a primer set specific for bBST-2A or bBST-2B, respectively. (B) Amino acid sequence alignment of bovine BST-2s, bBST-2A1, bBST-2A2 and bBST-2B, with human BST-2 (GenBank accession No. NM_004335). Asterisks show amino acid residues conserved in the BST-2A1 sequence, and bars indicate spaces. The predicted transmembrane domains (yellow), *N*-glycosylation sites (blue) and GPI-recognition motifs (red) are boxed. Posttranslational modifications were predicted using the TMHMM Server (http://www.cbs.dtu.dk/services/TMHMM), the NetNglyc 1.0 Server (http://www.cbs.dtu.dk/services/NetNGlyc) and the big-PI Predictor (http://mendel.imp.ac.at/gpi/gpi_server.html). (C) The maximum-likelihood phylogenetic tree of bBST-2s and human BST-2. The percent values were determined from 1000 repeats of fast bootstrapping using RAxML [34] are indicated at the branch junctions.

The bovine genome was predicted to contain two BST-2-like genes, BST-2A and BST-2B, located in close proximity on chromosome 7 [18], [37]. For molecular cloning of the complete coding regions, we designed primer sets specific for each of these bovine BST-2 (bBST-2) genes–bBST-2A and bBST-2B–and carried out RT-PCR using RNA extracted from a bovine cell line, MDBK cells, treated with the type I IFN, IFN-α, for 24 h. We successfully amplified bBST-2A and bBST-2B cDNAs by RT-PCR (Figure 1A). Interestingly, cDNA amplified for the bBST-2A gene consisted of two independent clones with different lengths and sequences. These were designated bBST-2A1 and bBST-2A2. The deduced amino acid sequences for bBST-2 clones are shown in Figure 1B. bBST-2A1 was seven amino acids longer than bBST-2A2 and had eight amino acid residues that were different from bBST-2A2. The degrees of amino acid sequence identity between bBST-2B and bBST-2A1 or bBST-2A2 were 78% or 77%, respectively. cDNA clones of bBST-2A1, bBST-2A2 and bBST-2B were also identified from the bovine trophoblast cell line BT-1 treated with type I IFN (data not shown). The nucleotide sequences of the coding region of bBST-2A1, bBST-2A2 and bBST-2B and the corresponding protein sequences have been deposited in DDBJ (AB698752 for bBST-2A1, AB698753 for bBST-2A2, and AB698754 for bBST-2B).

**Figure 2:**
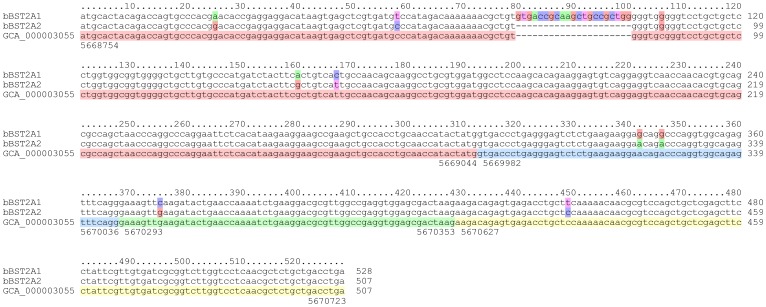
Multiple alignment of bBST-2A1, bBST-2A2 and the corresponding regions in the bovine genome. The coloured boxes of bBST-2A1 and bBST-2A2 indicate nucleotide differences. The coloured boxes in the genomic sequences (GenBank, GCA_000003055.3) show the exon structures. The numbers at the top or bottom of the alignment indicate the relative positions or the absolute genomic positions on chromosome 7, respectively.

The nucleotide sequence of bBST-2A2 determined in this study was almost identical to that from the genome data of *Bos taurus* (Figure 2). There is only one nucleotide difference between the sequences of bBST-2A2 and the genome data. The entire nucleotide sequences of bBST-2A1 and bBST-2A2 aligned except for an indel at positions 80–100 (21 nt, Figure 2). The aligned nucleotides varied at the following nine positions: 25, 58, 81, 160, 167, 342, 346, 375 and 449 (Figure 2). At least one nucleotide difference was observed in each of the four exons. In humans, on the other hand, indels and nucleotide sequence variations in BST-2 were only observed in the alternative exons (data not shown). Interestingly, eight of the nine nucleotide differences between bBST-2A1 and bBST-2A2 were nonsynonymous substitutions. Although these sequences were shown to be localized at the same genomic locus by BLAT, the indel region of bBST-2A1 was not identified in the genome (Figure 2). These observations can be explained by gene duplication occurring as a tandem repeat. If this is the case, the duplicated region can be missing due to misassembly. The strong selection on bBST-2A1 or bBST-2A2 also indicates that these are two distinct genes retained through neofunctionalisation. Indeed, the locus of bBST-2B is found to be very close to the genome [37], which can be also caused by tandem gene duplication. Thus, the genome analysis for *Bos taurus* suggested that bBST-2A1 and bBST-2A2 were not splicing variant isoforms.

### Posttranslational modification of bBST-2s

**Figure 3:**
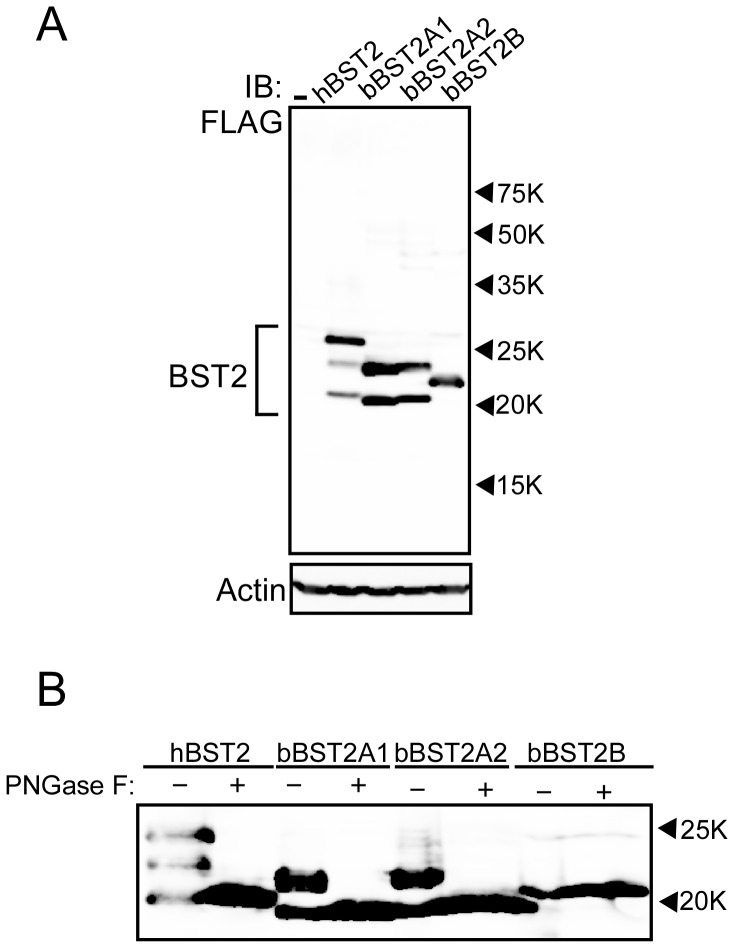
Posttranslational modifications for bovine BST-2s. (A) Expression plasmid for FLAG-tagged bBST-2A1, bBST-2A2 or bBST-2B was transfected into HEK 293T cells. Cells were dissolved 24 h after transfection and subjected to 12.5% SDS-PAGE. Western blotting was performed with anti-FLAG or anti-Actin antibody. (B) Bovine BST-2s expressed in HEK 293T cells were treated with (+) or without (−)PNGase F for de-*N*-glycosylated reaction, and then subjected to 12.5% SDS-PAGE. Reaction conditions with PNGase F were as described in the Materials and Methods. BST-2 was detected with anti-FLAG antibody by Western blotting analysis.

It has been reported that human BST-2 (hBST-2) has two *N*-linked glycosylation sites and a GPI-anchor [19]. As shown in Figure 1B, both bBST-2A1 and bBST-2A2 contain a putative *N*-linked glycosylation site and a GPI-anchoring motif, while bBST-2B has neither of them. To confirm the posttranslational modification of bBST-2s, we constructed the expression plasmids for bBST-2A1, bBST-2A2 and bBST-2B, which contain a FLAG-tag at the N-terminus. The expression plasmids were transfected into HEK293T cells and their expressions were analyzed by Western blot assay (Figure 3A). As previously reported, hBST-2 was detected as triplet bands, which indicate that the upper, middle, and lower bands of triplet forms corresponded to double-, single-, and non-glycosylated forms, respectively, [7], [16]. As expected, bBST-2A1 and bBST-2A2 were detected as double bands, while bBST-2B was detected as a single band, suggesting that bBST-2A1 and bBST-2A2 are glycosylated at a single site and bBST-2B is non-glycosylated. We further analyzed the glycosylation of bBST-2s by using PNGase F. As shown in Figure 3B, after treatment with PNGase F, the triplet bands of hBST-2 and the doublet bands of bBST-2A1 and bBST-2A2 converged on the lower single band, while bBST-2B remained as a single band, indicating that bBST-2A1 and bBST-2A2 are actually glycosylated and bBST-2B is non-glycosylated.

The position of the bBST-2B band was higher than those of the others despite its shorter amino acid sequence compared to hBST-2 and bBST-2A1 (Figures 1 and 3A). This suggested that hBST-2, bBST-2A1 and bBST-2A2 are cleaved at the GPI-anchoring signal and attached GPI anchor, while bBST-2B is neither cleaved nor possesses an attached GPI anchor due to the absence of a GPI-anchoring signal.

### Subcellular localization of bBST-2s

**Figure 4:**
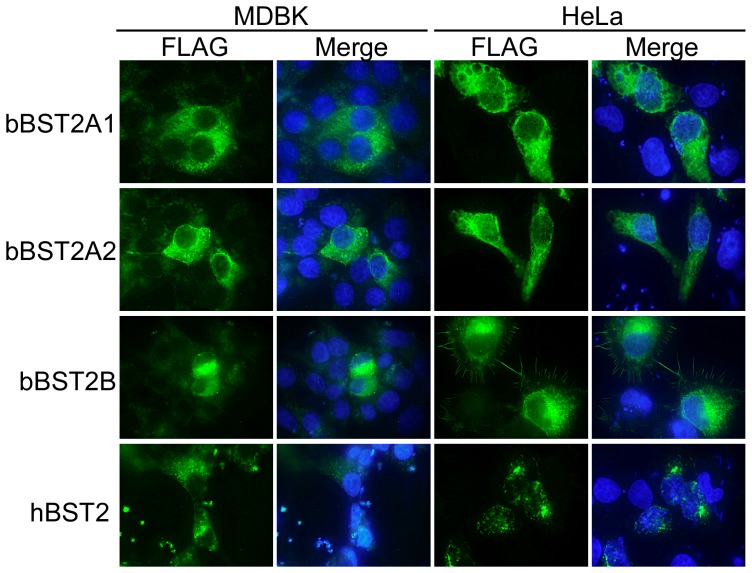
Subcellular localization of bovine BST-2s. FLAG-tagged bBST-2s expressed in MDBK cells (left two lines) and HeLa cells (right two lines) were stained with FITC-conjugated anti-FLAG antibody (showed green) and then observed by fluorescence microscopy. Nuclei stained with DAPI are shown in blue the merged panels.

We next examined the subcellular localization of bBST-2s by fluorescence microscopy. MDBK and HeLa cells expressing FLAG-tagged BST-2s were stained with FITC-conjugated anti-FLAG antibody (Figure 4). Both bBST-2A1 and bBST-2A2 showed a broad distribution in the cytoplasm of both MDBK and HeLa cells, while bBST-2B was mainly localized in the perinuclear compartment in both MDBK and HeLa cells.

**Figure 5:**
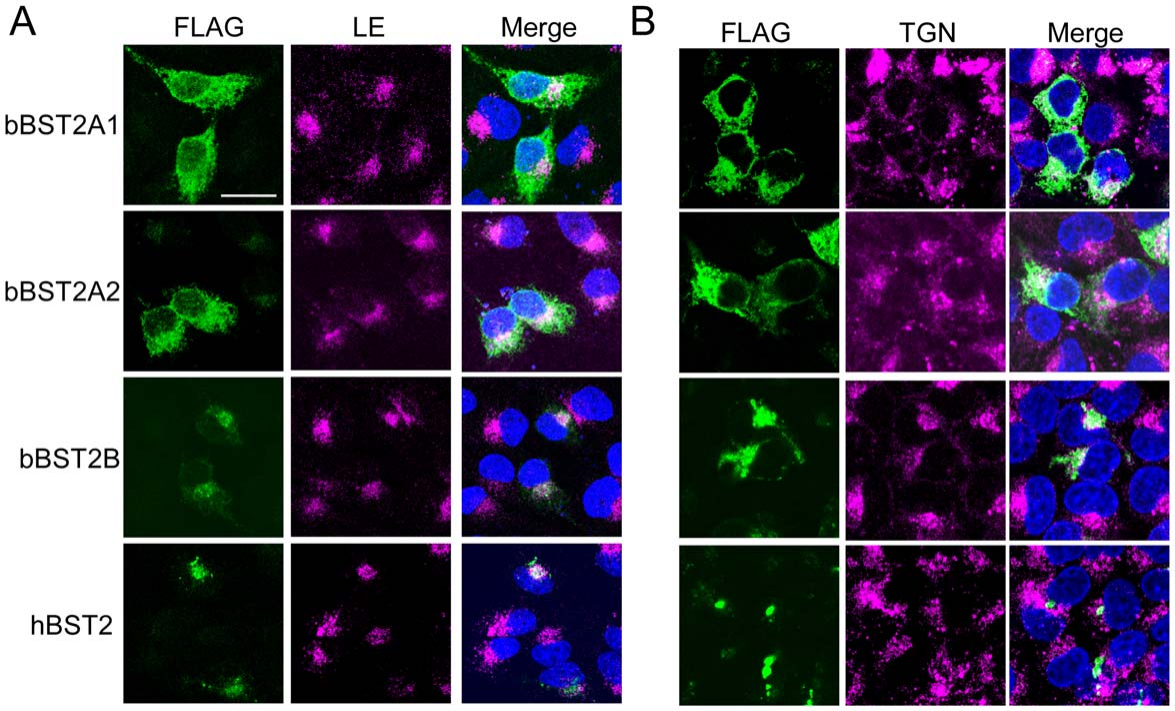
Co-localization of bovine BST-2 to the late-endosome (LE)/trans-Golgi network (TGN) compartments. FLAG-tagged bBST-2s expressed in HeLa cells were stained with FITC-conjugated anti-FLAG antibody (left panels) and then observed by confocal laser microscopy. LE or TGN was stained with anti-CD63 antibody (A) or anti-TGN46 antibody (B), respectively. Merged images of bBST-2s (green), organelle marker (red), and nuclei (blue) are shown in the right panels.

To further investigate the localization of bBST-2s, we examined the co-localization of bBST-2s with the late endosome (LE: stained with CD63 as an LE marker) or the trans-Golgi network (TGN: stained with TGN46 as a TGN marker) in HeLa cells using the confocal laser microscope (Figures 5A and B). hBST-2 was localized mainly in the LE and partially in the TGN as reported previously [19], [38]. The localization of bBST-2B was similar to that of hBST-2. Both bBST-2A1 and bBST-2A2 were localized preferentially in the LE and partially in the TGN, although they were broadly distributed in the cytoplasm.

### Antiviral activity of bBST-2s

It has been reported that hBST-2 has antiviral activities against a wide range of enveloped viruses [3], [7], [9]. To examine the antiviral activities of bBST-2s, we investigated the effect of exogenous expression of bBST-2s on the replication of BLV and VSV, which are pathogenic viruses for cattle.

**Figure 6:**
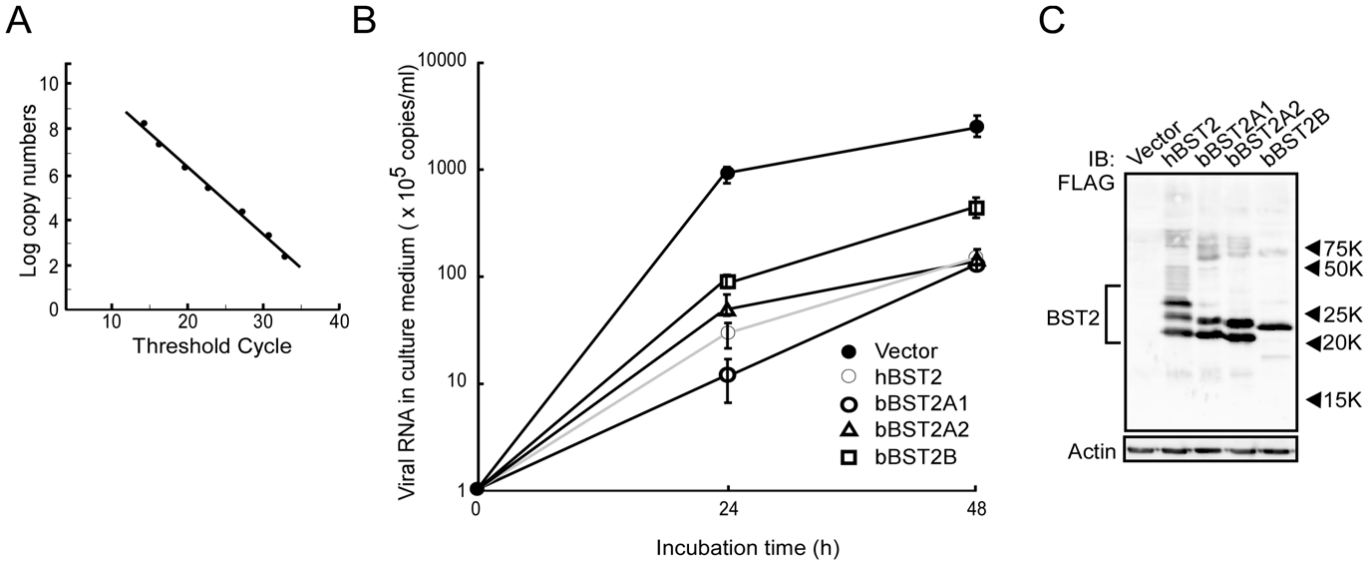
Antiviral activity of bovine BST-2s against BLV. (A) Standard curve of BLV-specific SYBR Green real-time RT-PCR used for the quantification of viral production was generated from the threshold cycle obtained against 10 fold serial dilutions of the plasmid as described in Materials and Methods. The assay was linear over a 7 log range from 2.4×10^2^–2.4×10^9^ copies. The coefficient of determination (r^2^) of the standard curve was 0.991. (B) Expression plasmid for bBST-2A1, bBST-2A2, bBST-2B or hBST-2 was transfected into FLK-BLV cells by electroporation. FLK-BLV cells expressing BST-2s were incubated for 24 h or 48 h. Culture supernatants were collected and virus production was determined by quantitative real-time RT-PCR. BLV productions were shown as copy numbers of viral RNA in culture supernatants (copies/ml). The experiments were conducted in triplicate, and the data are shown as the means ± standard deviation. (C) Cells were dissolved at 16 h after transfection and then subjected to 12.5% SDS-PAGE. Intracellular expression of BST-2s was confirmed by Western blotting using anti-FLAG antibody.

First, we examined the antiviral activities of bBST-2s against BLV. The expression plasmid for hBST-2 or bBST-2s was transfected into FLK-BLV cells constitutively producing BLV. At 12 h posttransfection, culture media were changed to fresh one. After incubation for 24 h or 48 h, BLV productions into culture media from cells were quantified by real-time RT-PCR with BLV specific primers. The real-time RT-PCR that we established in this study showed linear relationship for wide range of 2.4×10^2^–2.4×10^9^ copies (r^2^ = 0.991) (Figure 6A). The melting curve analysis revealed that the BLV-specific amplicon in this assay melts at 88.8°C. As shown in Figure 6B, BLV release from FLK-BLV cells significantly decreased by the expression of hBST-2 and bBST-2s. During 48 h incubation, bBST-2A1 and bBST-2A2 inhibited the BLV release to 4% of the control as well as hBST-2, while the inhibitory effect of bBST-2B on BLV release was weaker than those of the others. At 16 h after transfection, cell lysates were also analyzed by Western blotting to confirm the expression of hBST-2 or bBST-2s. As shown in Figure 6C, the expression levels of hBST-2, bBST-2A1, bBST-2A2 and bBST-2B in cells were similar.

**Figure 7:**
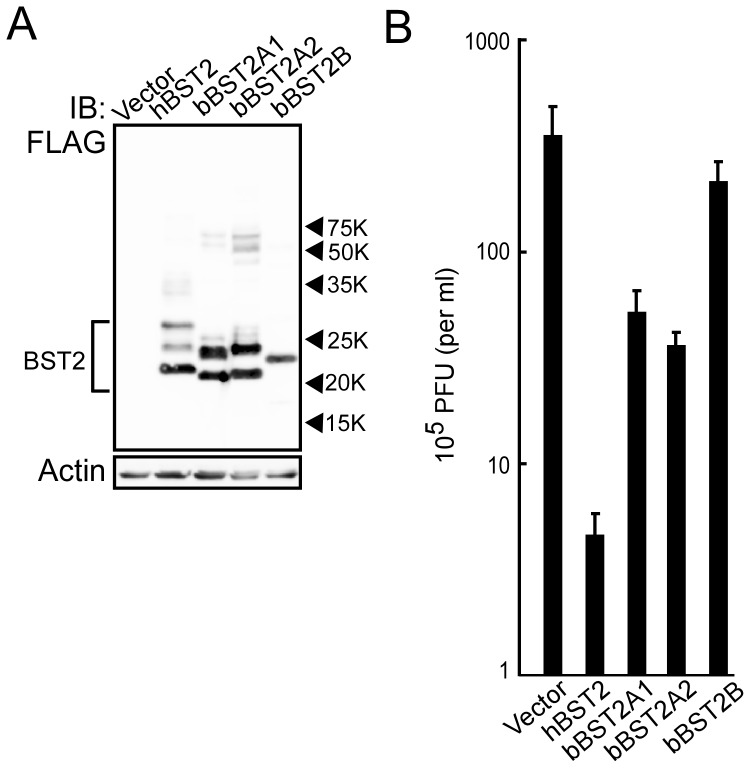
Antiviral activity of bovine BST-2s against VSV. Expression plasmid for bBST-2A1, bBST-2A2, bBST-2B or hBST-2 was transfected into MDCK cells by electroporation. (A) Cells were dissolved at 16 h after transfection and then subjected to 12.5% SDS-PAGE. Intracellular expression of BST-2s was confirmed by Western blotting using anti-FLAG antibody. (B) At 24 h after transfection, cells were infected with VSV at MOI of 0.001. At 12 h after infection, culture supernatants were collected and virus production was determined by plaque assay. The experiments were conducted in triplicate, and the data are shown as the means ± standard deviation.

Next, to examine the antiviral activities of bBST-2s, MDCK cells expressing bBST-2s or hBST-2 were infected with VSV at MOI of 0.001. At 12 h postinfection, virus production from cells was determined by plaque assay. Cell lysates were also analyzed by Western blotting to confirm the expression of bBST-2s or hBST-2. The expression levels of hBST-2, bBST-2A1, bBST-2A2 and bBST-2B in cells were similar (Figure 7A). As reported previously, hBST-2 significantly inhibited the production of VSV (Figure 7B) [9]. Both bBST-2A1 and bBST-2A2 also showed antiviral activity against VSV, although their activities were much weaker than that of hBST-2. On the other hand, bBST-2B had little effect on VSV production.

## Discussion

Previous analysis of the bovine genome database suggested that the bovine genome contains two BST-2-like genes, bBST-2A and bBST2-B, located in close proximity on chromosome 7 [18], [39]. BST-2 gene duplication has been already observed in sheep, and the paralogues are referred to as ovine BST-2A and BST2-B (oBST2A and oBST2B) [18]. Interestingly, in the present study, we identified three isoforms of bBST-2, designated as bBST-2A1, bBST-2A2 and bBST-2B. This BST-2 gene triplication in the genome has not been reported previously in other species. The degrees of amino acid sequence identity between oBST-2A and bBST-2A1 or bBST-2A2 were 86% or 85%, respectively. In addition, that between oBST-2B and bBST-2B was 86%. As shown in Figure 1C, the phylogenetic tree suggested that bBST-2As and bBST-2B are homologues of oBST-2A and oBST-2B, respectively.

BST-2 has been identified in several species, including human, monkeys, pig, mouse, cat and sheep [1], [2], [4], [11], [12], [13], [14], [15], [16], [17], [18]. All BST-2s from these species have been reported to possess antiviral activity. In addition, Arnaud *et al.* have reported that both oBST2A and oBST2B inhibited the production of sheep endogenous betaretrovirus (endogenous Jaagsiekte sheep retrovirus: enJSRV), although the antiviral activity of oBST2B was weaker than that of oBST2A [18]. Xu *et al.* previously reported that bBST-2 had antiviral activity against prototypic foamy virus, which is not bovine virus [26]. However, they identified only one isoform of BST-2 and examined its antiviral activity, since they did not notice that there are three isoforms of BST-2 in cattle. In this study, we examined the antiviral activities of three isoforms of bovine BST-2 against BLV and VSV. BLV is the bovine deltaretrovirus which cause enzootic bovine leucosis, while VSV is a member of the family Rhabdoviridae and causes an acute viral vesicular disease in cattle, horses and pigs. Both bBST-2A1 and bBST-2A2 showed apparent antiviral activities against both BLV and VSV (Figures 6 and 7). On the other hand, bBST-2B showed a weaker antiviral activity than bBST-2A1 and bBST-2A2 against BLV and a faint antiviral activity against VSV. bBST-2B mainly localized in the perinuclear compartment, and its subcellular localization was similar to that hBST-2, which has strong antiviral activity. bBST-2B does not possess the GPI-anchoring signal and therefore does not have an attached GPI anchor (Figures 1B and 3A). The absence of a GPI anchor on bBST2B would explain why the antiviral activity of bBST2B is very weak, as the GPI anchor has been reported to be critical for the antiviral activity of hBST2 [7], [9], [20].

All three isoforms of bBST-2 were greatly induced by treatment with type I IFN in bovine cells (Figure 1A). Both bBST2A-1 and bBST2A-2 may be involved in innate immunity, as these bBST2s have antiviral activity. It is likely that they have differential roles in host defence against viruses or other physiological functions. On the other hand, it is difficult to speculate on the physiological function of bBST-2B. However, it appears to have an important function as BST-2B is conserved in ruminants. To determine the physiological function of these bBST-2s, further detailed analyses of the *in vivo* expression of these genes are required.
